# Supplementary material for: Developing a multimodal therapy for glioblastoma using oncolytic virus delivering CD19 and EGFRvIII antigens and bi-specific CARs
Source: Nat Commun. 2026 Apr 9;17:4839. doi: 10.1038/s41467-026-71021-x (PMC13222885; doi:10.1038/s41467-026-71021-x)
Supplement: Supplementary file 1 — Supplementary Information [file 41467_2026_71021_MOESM1_ESM.pdf]

Supplementary Information

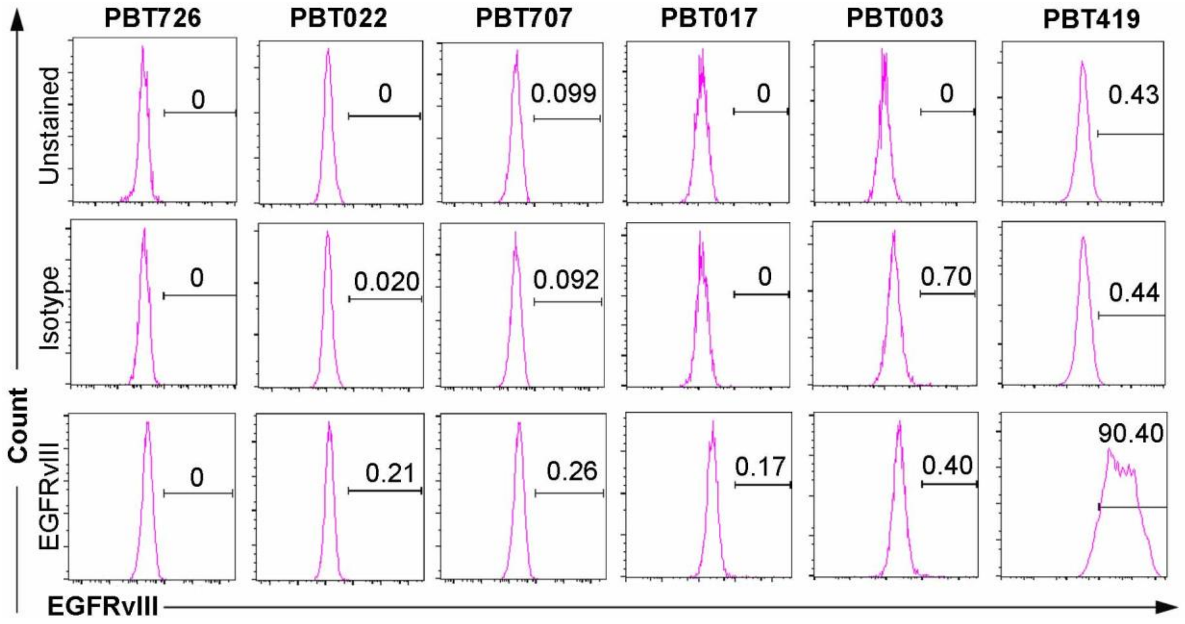

Fig. S1. **Endogenous EGFRvIII expression in patient-derived GBM cells as revealed by flow cytometry analysis.**

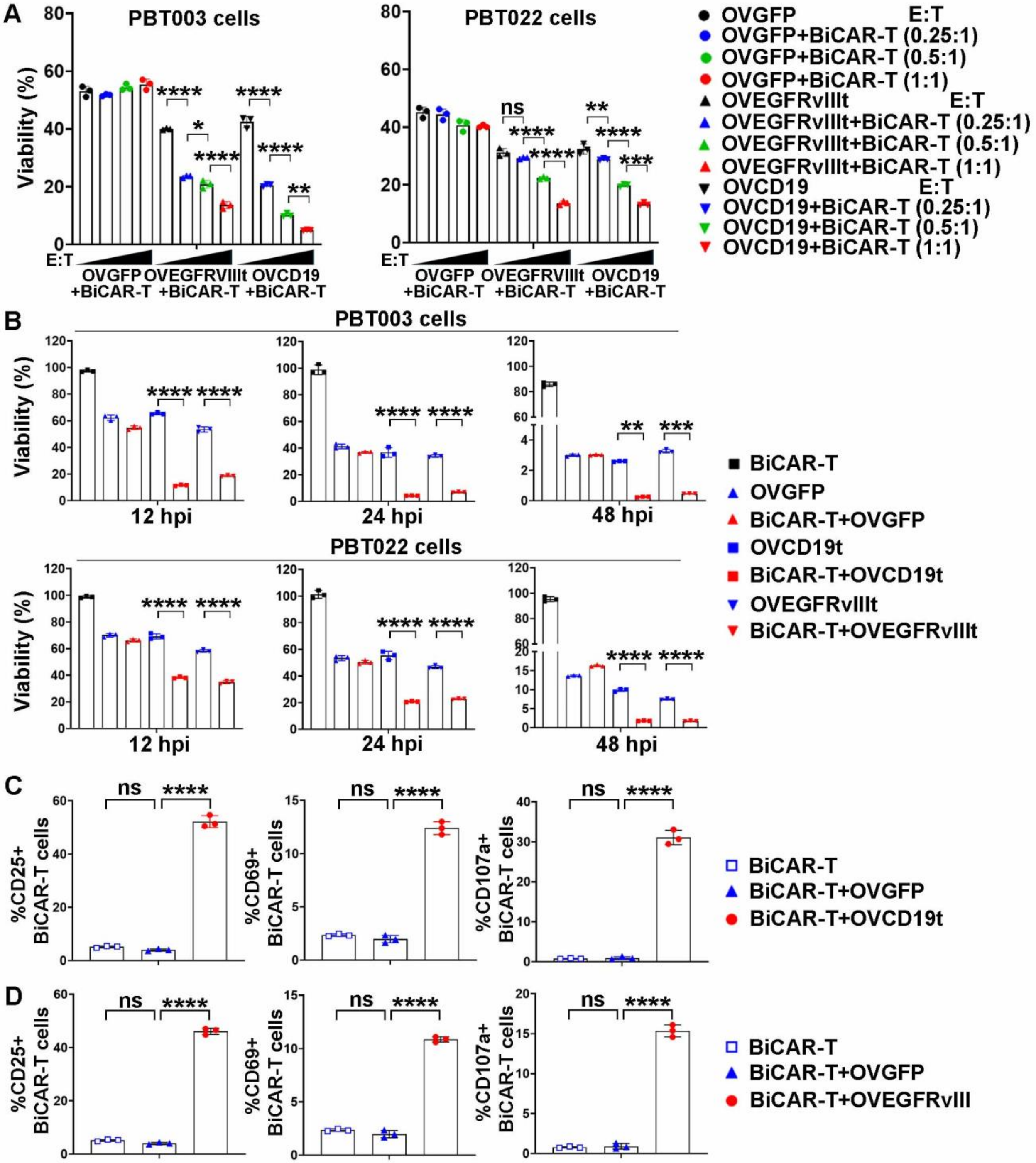

A
PBT003 cells
PBT022 cells
Viability (%)
E:T
OVGFP +BiCAR-T
OVEGFRVIIIt +BiCAR-T
OVCD19 +BiCAR-T
OVGFP E:T
OVGFP+BiCAR-T (0.25:1)
OVGFP+BiCAR-T (0.5:1)
OVGFP+BiCAR-T (1:1)
OVEGFRvIIIt E:T
OVEGFRvIIIt+BiCAR-T (0.25:1)
OVEGFRvIIIt+BiCAR-T (0.5:1)
OVEGFRvIIIt+BiCAR-T (1:1)
OVCD19 E:T
OVCD19+BiCAR-T (0.25:1)
OVCD19+BiCAR-T (0.5:1)
OVCD19+BiCAR-T (1:1)
B
PBT003 cells
PBT022 cells
12 hpi
24 hpi
48 hpi
BiCAR-T
OVGFP
BiCAR-T+OVGFP
OVCD19t
BiCAR-T+OVCD19t
OVEGFRvIIIt
BiCAR-T+OVEGFRvIIIt
C
%CD25+ BiCAR-T cells
%CD69+ BiCAR-T cells
%CD107a+ BiCAR-T cells
BiCAR-T
BiCAR-T+OVGFP
BiCAR-T+OVCD19t
D
%CD25+ BiCAR-T cells
%CD69+ BiCAR-T cells
%CD107a+ BiCAR-T cells
BiCAR-T
BiCAR-T+OVGFP
BiCAR-T+OVEGFRvIII

Fig. S2. BiCAR-T cells are activated by CD19t- or EGFRvIIIt-encoding oncolytic virus in vitro. (**A** and **B**) BiCAR-T-mediated cytotoxicity was measured in PBT003 and PBT022 GBM cells infected with CD19t or EGFRvIIIt-encoding oncolytic virus using a luciferase reporter assay. For A: OV-infected GBM cells were co-cultured with BiCAR-T cells at different E:T ratio for 24 h. n = 3 cell culture replicates. PBT003: *p = 0.0371, **p = 0.0013, ****p < 0.0001. PBT022: **p = 0.0093, ***p = 0.0002, ****p < 0.0001. For B: Infected GBM cells were co-cultured with BiCAR-T cells at a 1:1 E/T ratio for 12, 24, or 48 hours. n = 3. **p = 0.0084 (OVCD19t versus BiCAR-T+OVCD19t), **p = 0.0019 (OVEGFRvIIIt versus BiCAR-T+OVEGFRvIIIt), ****p < 0.0001. (**C** and **D**) Enhanced activation of BiCAR-T cells as revealed by an increased percentage of CD25-positive, CD69-positive, or CD107a-positive cells detected by flow cytometry analysis. Because the experiments for Fig. S2C and S2D were performed together, the BiCAR-T and BiCAR-T+OVGFP control groups are shared in these two panels. n = 3 cell culture replicates. For panels A-D, data are presented as mean ± SD and were analyzed by one-way ANOVA with Tukey multiple comparisons test. ns means no significant difference. Source data are provided in the Source Data file.

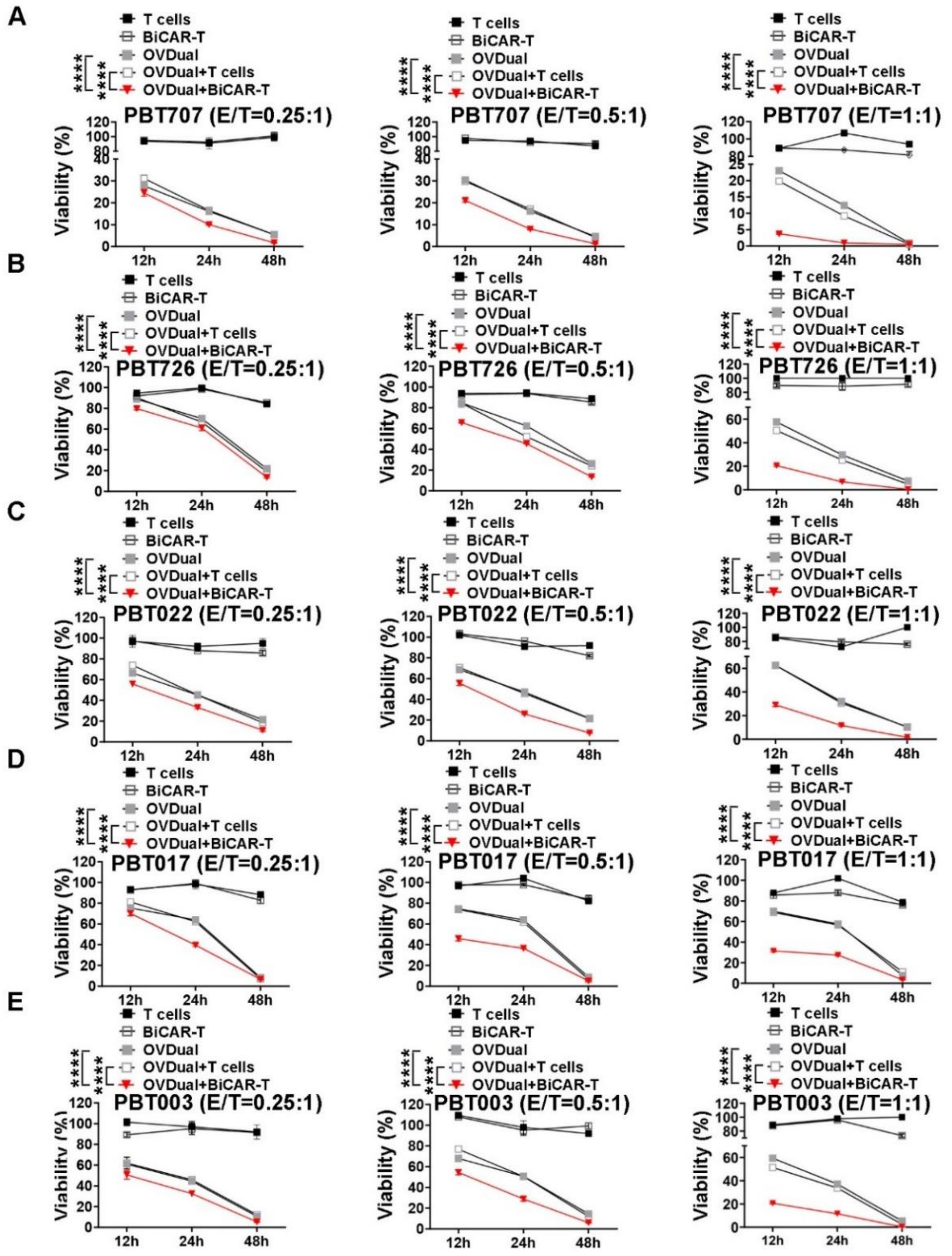

Fig. S3. **Tumor-killing efficacy of OVDual in combination with BiCAR-T cells targeting different patient-derived GBM cells.** (**A–E**) GBM cells (PBT707, PBT726, PBT022, PBT017, PBT003) were infected ± OVDual (MOI = 1, 5 h) and then treated ± control T or BiCAR-T cells at different E/T ratios. The data of E/T ratio at 1 are from Fig. 2B and included here to show the dose response. Cell viability was measured by a luciferase reporter assay at 12, 24, or 48 h after control T or BiCAR-T treatment. n = 3 cell culture replicates. Data are presented as mean ± SD and were analyzed by two-way ANOVA followed by Tukey's multiple comparisons test. ****p < 0.0001. Source data are provided in the Source Data file.

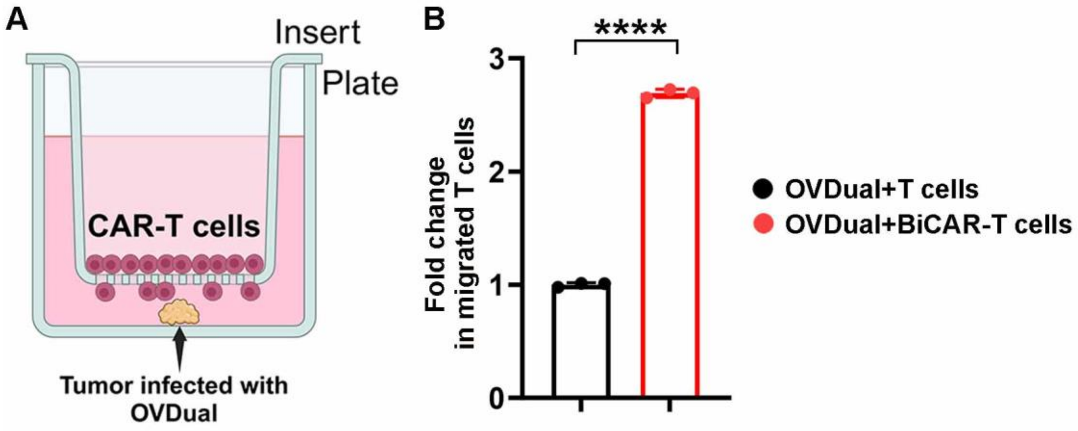

Fig. S4. **BiCAR-T cells migrate more effectively towards OVDual-infected GBM cells than control T cells.** (**A**) A schematic of the transwell assay to assess BiCAR-T cell migration into OVDual-infected 3D tumor spheroids. T cells without CAR were included as a control (generated using BioRender). (**B**) Quantification of BiCAR-T cell migration towards OVDual-infected 3D tumor spheroids. n = 3 cell culture replicates. Data are presented as mean ± SD and were analyzed with two-tailed unpaired t tests. ****$p < 0.0001$. Source data are provided in the Source Data file.

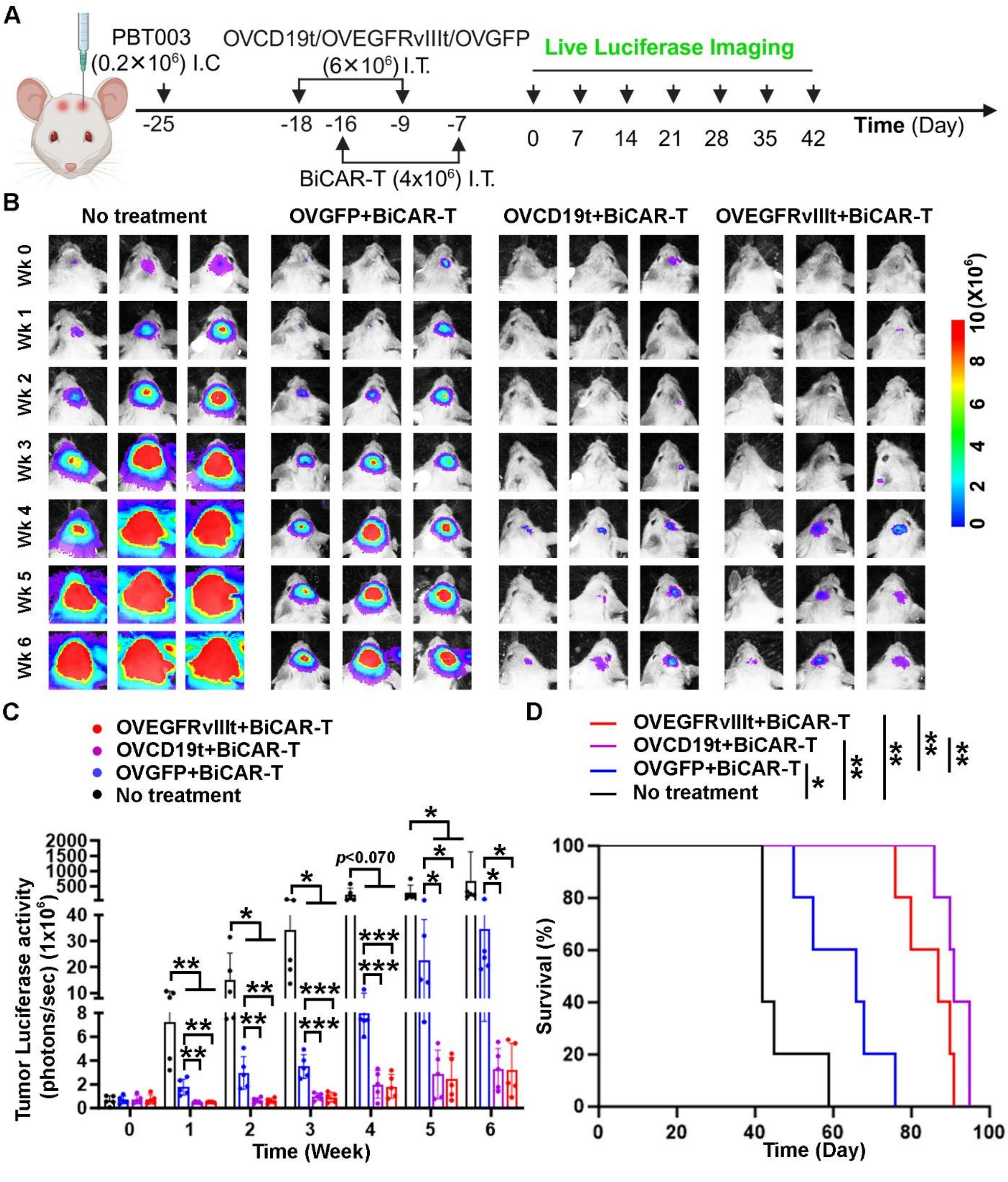
A
PBT003
(0.2×10^6) I.C
OVCD19t/OVEGFRvIIIt/OVGFP
(6×10^6) I.T.
Live Luciferase Imaging
-25
-18
-16
-9
-7
0
7
14
21
28
35
42
Time (Day)
BiCAR-T (4x10^6) I.T.
B
No treatment
OVGFP+BiCAR-T
OVCD19t+BiCAR-T
OVEGFRvIIIt+BiCAR-T
Wk 0
Wk 1
Wk 2
Wk 3
Wk 4
Wk 5
Wk 6
10(X10^6)
C
OVEGFRvIIIt+BiCAR-T
OVCD19t+BiCAR-T
OVGFP+BiCAR-T
No treatment
Tumor Luciferase activity (photons/sec) (1x10^6)
p<0.070
Time (Week)
D
OVEGFRvIIIt+BiCAR-T
OVCD19t+BiCAR-T
OVGFP+BiCAR-T
No treatment
Survival (%)
Time (Day)

Fig. S5. **BiCAR-T cells are effectively activated by CD19t- or EGFRvIIIt-encoding oncolytic virus in a xenograft GBM mouse model.** (**A**) A schematic of GBM cell (PBT003-luc)-bearing mice treated with OVCD19t, OVEGFRvIIIt or OVGFP in combination with BiCAR-T cells. I.C.: intracranial; I.T.: intratumoral (generated using BioRender). (**B**) Bioluminescence images of brain tumors in NSG mice. (**C**) Quantification of the bioluminescence intensity of tumors in mice brains. n = 5 mice per group. Data are presented as mean ± SD and were analyzed by two-tailed unpaired t tests. Week1: ** p = 0.0077 (OVGFP+BiCAR-T vs no treatment), **p = 0.0022 (OVCD19t+BiCAR-T vs no treatment), **p = 0.0022 (OVEGFRvIIIt+BiCAR-T vs no treatment), **p = 0.0033 (OVCD19t+BiCAR-T vs OVGFP+BiCAR-T), **p = 0.0034 (OVEGFRvIIIt+BiCAR-T vs OVGFP+BiCAR-T). Week2: *p = 0.0296 (OVGFP+BiCAR-T vs no treatment), *p = 0.0131 (OVCD19t+BiCAR-T vs no treatment), *p = 0.0130 (OVEGFRvIIIt+BiCAR-T vs no treatment), **p = 0.0061 (OVCD19t+BiCAR-T vs OVGFP+BiCAR-T), **p = 0.0055 (OVEGFRvIIIt+BiCAR-T vs OVGFP+BiCAR-T). Week3: *p = 0.0212 (OVGFP+BiCAR-T vs no treatment), *p = 0.0148 (OVCD19t+BiCAR-T vs no treatment), *p = 0.0147 (OVEGFRvIIIt+BiCAR-T vs no treatment), ***p = 0.0006 (OVCD19t+BiCAR-T vs OVGFP+BiCAR-T), ***p = 0.0007 (OVEGFRvIIIt+BiCAR-T vs OVGFP+BiCAR-T). Week4: ***p = 0.0005 (OVCD19t+BiCAR-T vs OVGFP+BiCAR-T), ***p = 0.0003 (OVEGFRvIIIt+BiCAR-T vs OVGFP+BiCAR-T). Week5: *p = 0.0462 (OVCD19t+BiCAR-T vs no treatment), *p = 0.0460 (OVEGFRvIIIt+BiCAR-T vs no treatment), *p = 0.0215 (OVCD19t+BiCAR-T vs OVGFP+BiCAR-T), *p = 0.0197 (OVEGFRvIIIt+BiCAR-T vs OVGFP+BiCAR-T). Week6: *p = 0.0335 (OVCD19t+BiCAR-T vs OVGFP+BiCAR-T), *p = 0.0334 (OVEGFRvIIIt+BiCAR-T vs OVGFP+BiCAR-T). (**D**) The survival of GBM tumor-bearing mice. n = 5 mice per group. Statistical analysis was performed by log-rank test, *p = 0.0214 (OVGFP+BiCAR-T vs no treatment), **p = 0.0015 (OVCD19t+BiCAR-T vs no treatment), **p = 0.0015 (OVEGFRvIIIt+BiCAR-T vs no treatment), **p = 0.0018 (OVCD19t+BiCAR-T vs OVGFP+BiCAR-T), **p = 0.0044 (OVEGFRvIIIt+BiCAR-T vs OVGFP+BiCAR-T). Source data are provided in the Source Data file.

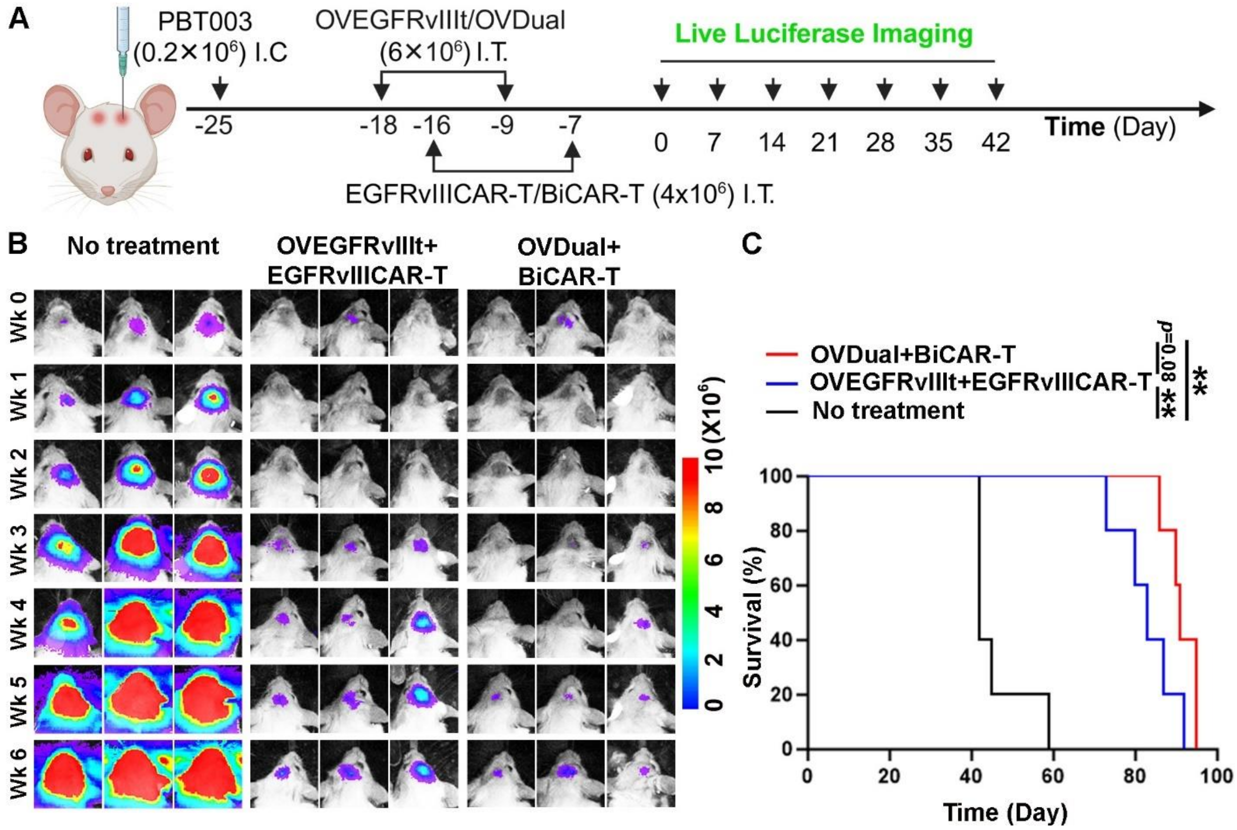

Fig. S6. **BiCAR-T and EGFRvIIICAR-T cell-mediated tumor killing in a xenograft GBM mouse model.** (**A**) A schematic of GBM cell (PBT003-luc)-bearing mice treated with OVDual plus BiCAR-T or OVEGFRvIIIt plus EGFRvIIICAR-T cells. I.C.: intracranial; I.T.: intratumoral (generated using BioRender). (**B**) Bioluminescence images of brain tumors in NSG mice transplanted with GBM cells treated with OVDual plus BiCAR-T or OVEGFRvIIIt plus EGFRvIIICAR-T or received no treatment. (**C**) The survival of GBM tumor bearing mice treated with OVDual plus BiCAR-T or OVEGFRvIIIt plus EGFRvIIICAR-T or received no treatment. n = 5 mice per group. Statistical analysis was performed by Log-rank test. **p = 0.0015 (OVEGFRvIIIt plus EGFRvIIICAR-T vs no treatment), **p = 0.0015 (OVDual plus BiCAR-T vs no treatment). Source data are provided in the Source Data file.

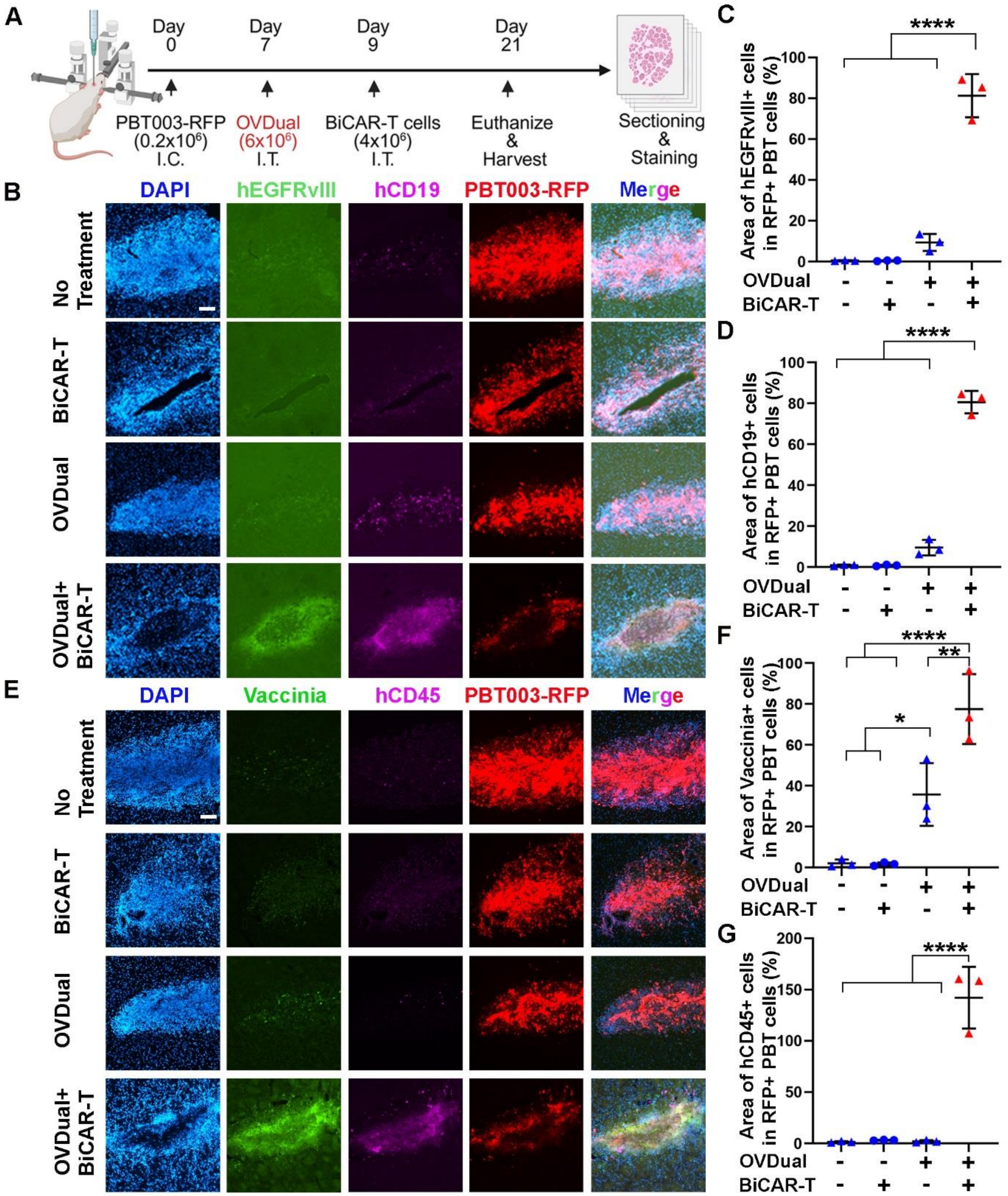

A
Day 0
Day 7
Day 9
Day 21
PBT003-RFP (0.2x10^6) I.C.
OVDual (6x10^6) I.T.
BiCAR-T cells (4x10^6) I.T.
Euthanize & Harvest
Sectioning & Staining
B
DAPI
hEGFRvIII
hCD19
PBT003-RFP
Merge
No Treatment
BiCAR-T
OVDual
OVDual+ BiCAR-T
C
Area of hEGFRvIII+ cells in RFP+ PBT cells (%)
****
OVDual
BiCAR-T
D
Area of hCD19+ cells in RFP+ PBT cells (%)
****
E
DAPI
Vaccinia
hCD45
PBT003-RFP
Merge
F
Area of Vaccinia+ cells in RFP+ PBT cells (%)
****
**
*
G
Area of hCD45+ cells in RFP+ PBT cells (%)
****

Fig. S7. **OVDual plus BiCAR-T treatment effectively modulates the tumor microenvironment. (A)** A schematic of the experimental procedure (generated using BioRender). (**B**) Representative images showing human EGFRvIII (hEGFRvIII)-positive and human CD19 (hCD19)-positive cells revealed by immunostaining of brain sections from mice received different treatments. Scale bar, 100 μm. (**C** and **D**) Quantification of the percentage of hEGFRvIII-positive area within the RFP-positive tumor area (C), and the percentage of hCD19-positive area within the RFP-positive tumor area (D). (**E**) Representative images showing the presence of OV by vaccinia viral protein staining and the presence of BiCAR-T cells by human CD45 (hCD45) staining of brain sections from mice received different treatments. Scale bar, 100 μm. (**F** and **G**) Quantification of the percentage of OV-targeted RFP-positive area within the RFP-positive tumor area (F), and the percentage of hCD45-positive T cell area within the RFP-positive tumor area (G). For panels C, D, F and G, n = 3 mice for each group, data from three brain slices were averaged to generate a single biological replicate, data are presented as mean ± SD and were analyzed by one-way ANOVA with Tukey's multiple comparisons test. *p = 0.0247, **p = 0.005, and ****p < 0.0001. Source data are provided in the Source Data file.

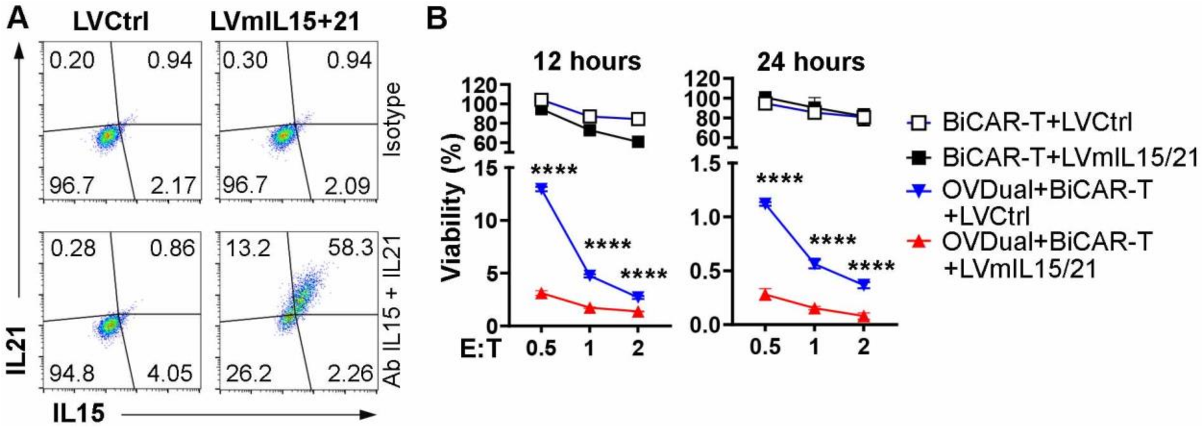

Fig. S8. GBM cells infected with a lentiviral vector encoding membrane-bound IL15 and IL21 (LVmIL15/21) enhance the cytotoxicity of BiCAR-T cells in vitro. (**A**) Representative flow cytometry plots showing the expression of IL15 and IL21 on the surface of GBM cells (PBT003) transduced with LVmIL15/21. GBM cells were transduced with the control lentiviral vector (LVCtrl) or LVmIL15/21 and analyzed by flow cytometry using anti-IL15 and anti-IL21 antibodies. (**B**) LVCtrl or LVmIL15/21-transduced GBM cells (PBT003) were infected with OVDual and then treated with BiCAR-T cells for 12 h or 24 h at various E:T ratios. Cells were collected at the indicated time points and analyzed using a luciferase reporter assay. n = 3 cell culture replicates. Data are presented as mean ± SD and were analyzed by two-way ANOVA with Tukey's multiple comparisons test. ****$p < 0.0001$. Source data are provided in the Source Data file.

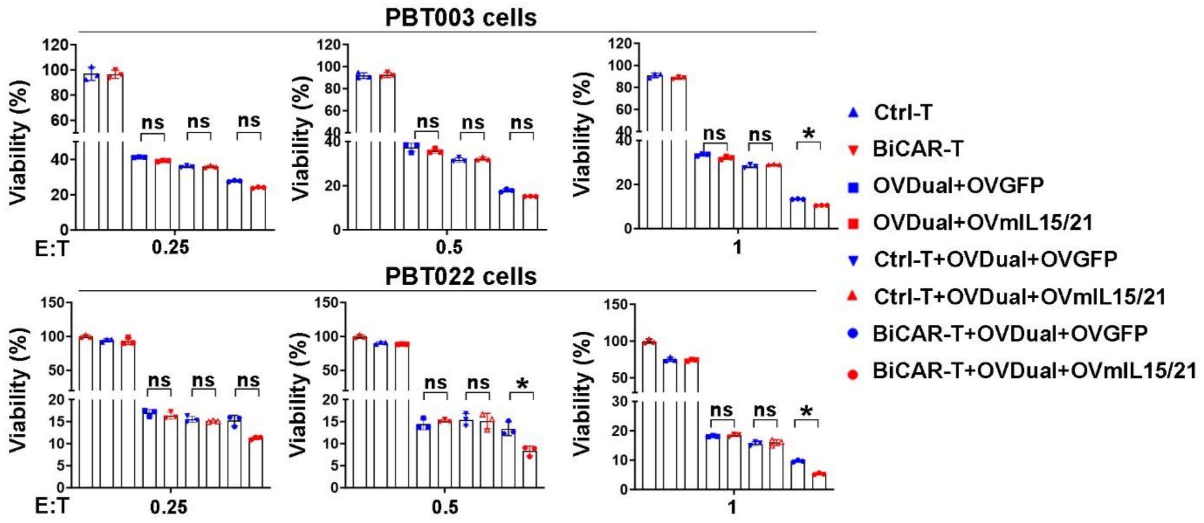

Fig. S9. OVmIL15/21-boosted BiCAR-T cytotoxicity is dependent on CAR-antigen engagement. Control T (Ctrl-T) or BiCAR-T-mediated cytotoxicity against PBT003 and PBT022 GBM cells infected with OVDual+OVGFP or OVDual+OVmIL15/21. GBM cells infected with the indicated OV combination were treated with Ctrl-T or BiCAR-T cells at different E:T ratios for 12, 24, or 48 h. n = 3 cell culture replicates. Data are presented as mean ± SD and were analyzed by One-way ANOVA with Tukey's multiple comparisons test. PBT003: *p = 0.0313 (E: T = 1); PBT022: *p = 0.0128 (E: T = 0.5), *p = 0.0474 (E: T = 1), and ns means no significant difference. Source data are provided in the Source Data file.

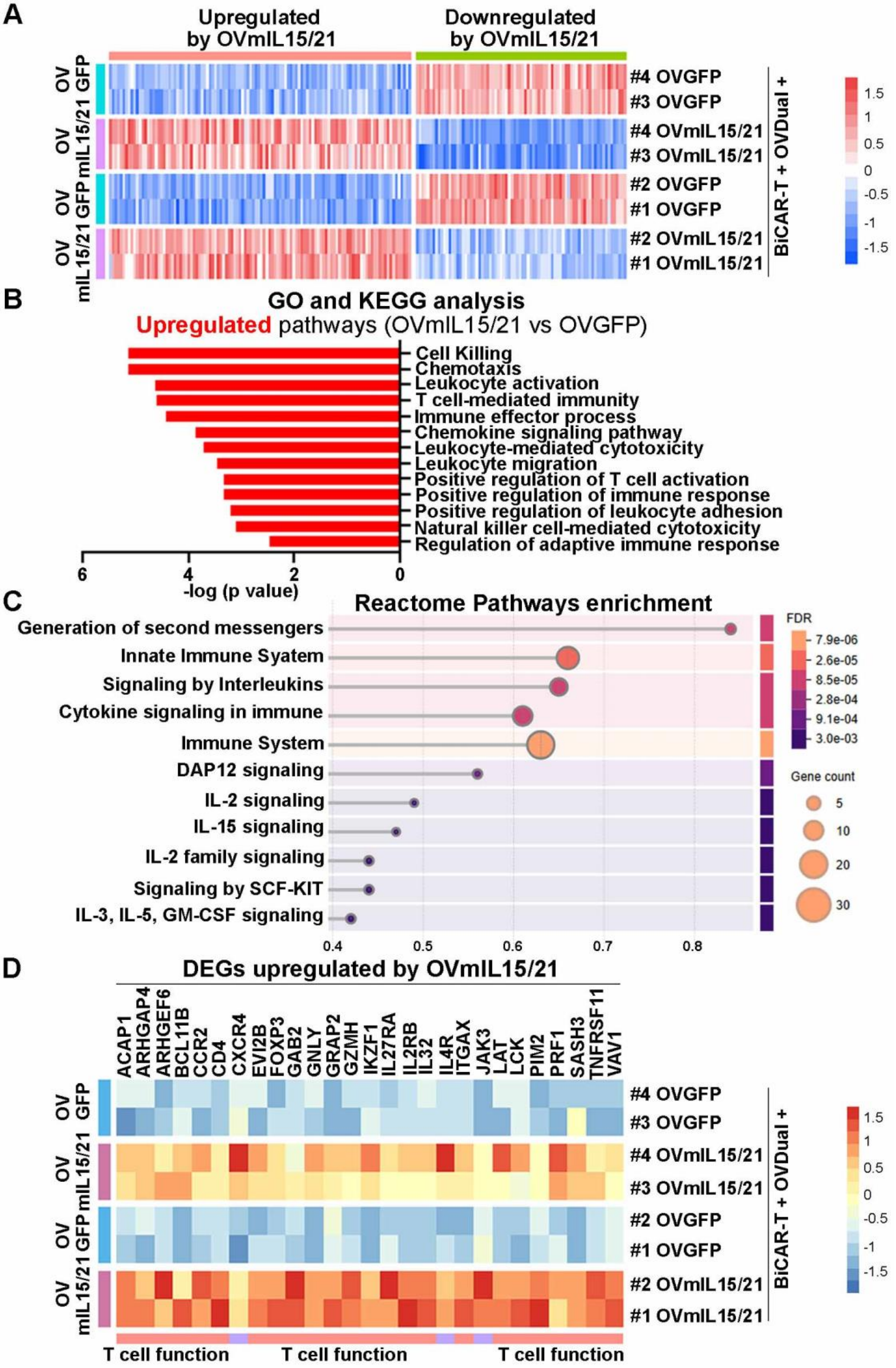
A
Upregulated by OVmIL15/21
Downregulated by OVmIL15/21
OV GFP
OV mIL15/21
#4 OVGFP
#3 OVGFP
#4 OVmIL15/21
#3 OVmIL15/21
#2 OVGFP
#1 OVGFP
#2 OVmIL15/21
#1 OVmIL15/21
BiCAR-T + OVDual +
1.5
1
0.5
0
-0.5
-1
-1.5
B
GO and KEGG analysis
Upregulated pathways (OVmIL15/21 vs OVGFP)
Cell Killing
Chemotaxis
Leukocyte activation
T cell-mediated immunity
Immune effector process
Chemokine signaling pathway
Leukocyte-mediated cytotoxicity
Leukocyte migration
Positive regulation of T cell activation
Positive regulation of immune response
Positive regulation of leukocyte adhesion
Natural killer cell-mediated cytotoxicity
Regulation of adaptive immune response
6 4 2 0
-log (p value)
C
Reactome Pathways enrichment
Generation of second messengers
Innate Immune Syatem
Signaling by Interleukins
Cytokine signaling in immune
Immune System
DAP12 signaling
IL-2 signaling
IL-15 signaling
IL-2 family signaling
Signaling by SCF-KIT
IL-3, IL-5, GM-CSF signaling
0.4 0.5 0.6 0.7 0.8
FDR
7.9e-06
2.6e-05
8.5e-05
2.8e-04
9.1e-04
3.0e-03
Gene count
5
10
20
30
D
DEGs upregulated by OVmIL15/21
ACAP1 ARHGAP4 ARHGEF6 BCL11B CCR2 CD4 CXCR4 EVI2B FOXP3 GAB2 GNLY GRAP2 GZMH IKZF1 IL27RA IL2RB IL32 IL4R ITGAX JAK3 LAT LCK PIM2 PRF1 SASH3 TNFRSF11 VAV1
#4 OVGFP
#3 OVGFP
#4 OVmIL15/21
#3 OVmIL15/21
#2 OVGFP
#1 OVGFP
#2 OVmIL15/21
#1 OVmIL15/21
BiCAR-T + OVDual +
1.5
1
0.5
0
-0.5
-1
-1.5
T cell function
T cell function
T cell function

Figure S10. Activation of downstream signaling pathways in BiCAR-T cells cocultured with GBM cells infected with OVmIL15/21 and OVDual. Total RNA was collected from BiCAR-T cells cocultured with OVDual and OVmIL15/21-infected PBT003 (#1 and #2) or PBT707 (#3 and #4) cells, or BiCAR-T cells cocultured with OVDual and OVGFP-infected PBT003 (#1 and #2) or PBT707 (#3 and #4) cells. All samples were collected 48 h after OV and BiCAR-T treatment, a time point at which GBM cells were fully eliminated. (**A**) Heatmap showing all differentially expressed genes (DEGs) with statistical significance (padj<0.05) in BiCAR-T cells cocultured with OVDual+OVmIL15/21 versus OVDual+OVGFP-infected GBM cells by RNA sequencing (RNAseq) analysis. (**B**) A bar graph of KEGG pathways and GO terms associated with upregulated genes (padj<0.05 and |FC|>1.5) in BiCAR-T cells cocultured with OVDual+OVmIL15/21-infected GBM cells versus BiCAR-T cells cocultured with OVDual+OVGFP-infected GBM cells. (**C**) A bar graph of Reactome enrichment pathways associated with upregulated genes (padj<0.05) in BiCAR-T cells cocultured with OVDual and OVmIL15/21-infected GBM cells versus BiCAR-T cells cocultured with OVDual and OVGFP-infected GBM cells. (**D**) The expression of specific DEGs (padj<0.05), including DEGs related to T cell function (labeled by pink bar), shown in a targeted heatmap.

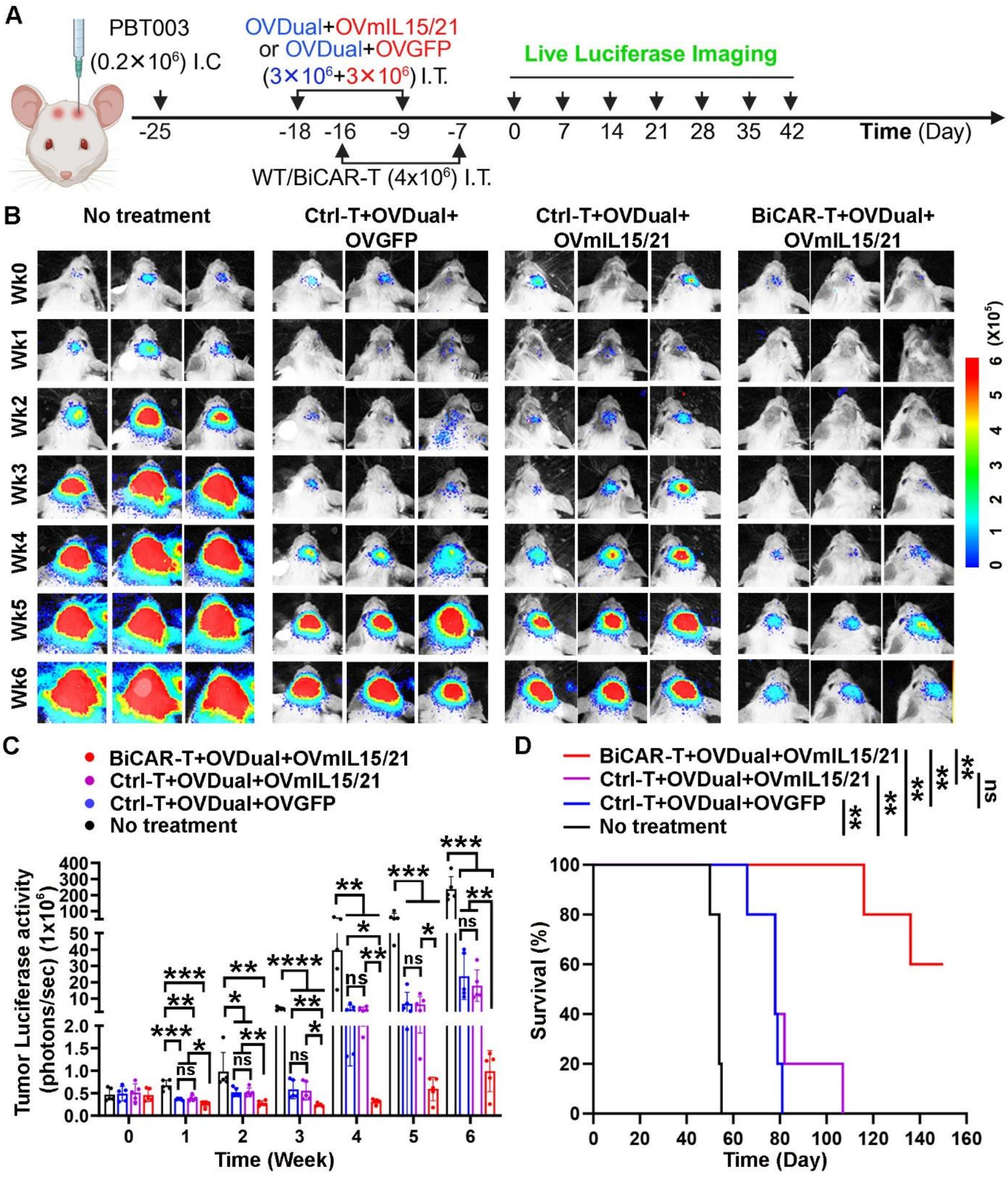

A
PBT003
(0.2×10^6) I.C
OVDual+OVmIL15/21
or OVDual+OVGFP
(3×10^6+3×10^6) I.T.
Live Luciferase Imaging
-25
-18
-16
-9
-7
0
7
14
21
28
35
42
Time (Day)
WT/BiCAR-T (4x10^6) I.T.
B
No treatment
Ctrl-T+OVDual+OVGFP
Ctrl-T+OVDual+OVmIL15/21
BiCAR-T+OVDual+OVmIL15/21
Wk0
Wk1
Wk2
Wk3
Wk4
Wk5
Wk6
(X10^5)
C
BiCAR-T+OVDual+OVmIL15/21
Ctrl-T+OVDual+OVmIL15/21
Ctrl-T+OVDual+OVGFP
No treatment
Tumor Luciferase activity (photons/sec) (1x10^6)
Time (Week)
D
BiCAR-T+OVDual+OVmIL15/21
Ctrl-T+OVDual+OVmIL15/21
Ctrl-T+OVDual+OVGFP
No treatment
Survival (%)
Time (Day)

Fig. S11. **OVmIL15/21 boosts BiCAR-T anti-tumor effect in a CAR-dependent manner.** (**A**) A schematic of GBM cell (PBT003-luc)-bearing mice treated with OVDual + OVmIL15/21 or OVDual plus OVGFP, followed by control T (Ctrl-T) or BiCAR-T cells. I.C.: intracranial; I.T.: intratumoral (generated using BioRender). (**B**) Bioluminescence images of brain tumors in GBM cell-bearing mice. (**C**) Quantification of the bioluminescence intensity of tumors after indicated treatments. n = 5 mice per group. Data are presented as mean ± SD and were analyzed by two-tailed unpaired t tests. Week1: ***p = 0.0003, **p = 0.0011, ***p = 0.0001 (Ctrl-T+OVDual+OVGFP, Ctrl-T+OVDual+OVmIL15/21, or BiCAR-T+OVDual+OVmIL15/21 vs no treatment); *p = 0.0105, *p = 0.0176 (BiCAR-T+OVDual+OVmIL15/21 vs Ctrl-T+OVDual+OVGFP or Ctrl-T+OVDual+OVmIL15/21). Week2: *p = 0.0474, *p = 0.0471, **p = 0.0065 (Ctrl-T+OVDual+OVGFP, Ctrl-T+OVDual+OVmIL15/21, or BiCAR-T+OVDual+OVmIL15/21 vs no treatment); **p = 0.0014 (BiCAR-T+OVDual+OVmIL15/21 vs Ctrl-T+OVDual+OVGFP or Ctrl-T+OVDual+OVmIL15/21). Week3: **p = 0.0066, *p = 0.0102 (BiCAR-T+OVDual+OVmIL15/21 vs Ctrl-T+OVDual+OVGFP or Ctrl-T+OVDual+OVmIL15/21). ****p < 0.0001. Week4: **p = 0.0028, **p = 0.0026, **p = 0.0016 (Ctrl-T+OVDual+OVGFP, Ctrl-T+OVDual+OVmIL15/21, or BiCAR-T+OVDual+OVmIL15/21 vs no treatment); *p = 0.0184, **p = 0.0013 (BiCAR-T+OVDual+OVmIL15/21 vs Ctrl-T+OVDual+OVGFP or Ctrl-T+OVDual+OVmIL15/21). Week5: ***p = 0.0008, ***p = 0.0007, ***p = 0.0003 (Ctrl-T+OVDual+OVGFP, Ctrl-T+OVDual+OVmIL15/21, or BiCAR-T+OVDual+OVmIL15/21 vs no treatment); *p = 0.0226 (BiCAR-T+OVDual+OVmIL15/21 vs Ctrl-T+OVDual+OVmIL15/21). Week6: ***p = 0.0003, ***p = 0.0002, ***p = 0.0001 (Ctrl-T+OVDual+OVGFP, Ctrl-T+OVDual+OVmIL15/21, or BiCAR-T+OVDual+OVmIL15/21 vs no treatment); **p = 0.0069, **p = 0.0042 (BiCAR-T+OVDual+OVmIL15/21 vs Ctrl-T+OVDual+OVGFP or Ctrl-T+OVDual+OVmIL15/21). ns means no significant difference. (**D**) The survival of GBM tumor bearing mice after indicated treatments. n = 5 mice per group. Data were analyzed by log-rank test. **p = 0.0020 (Ctrl-T+OVDual+OVGFP, Ctrl-T+OVDual+OVmIL15/21, or BiCAR-T+OVDual+OVmIL15/21 vs no treatment); **p = 0.0018 (BiCAR-T+OVDual+OVmIL15/21 vs Ctrl-T+OVDual+OVGFP or Ctrl-T+OVDual+OVmIL15/21). ns means no significant difference. Source data are provided in the Source Data file.

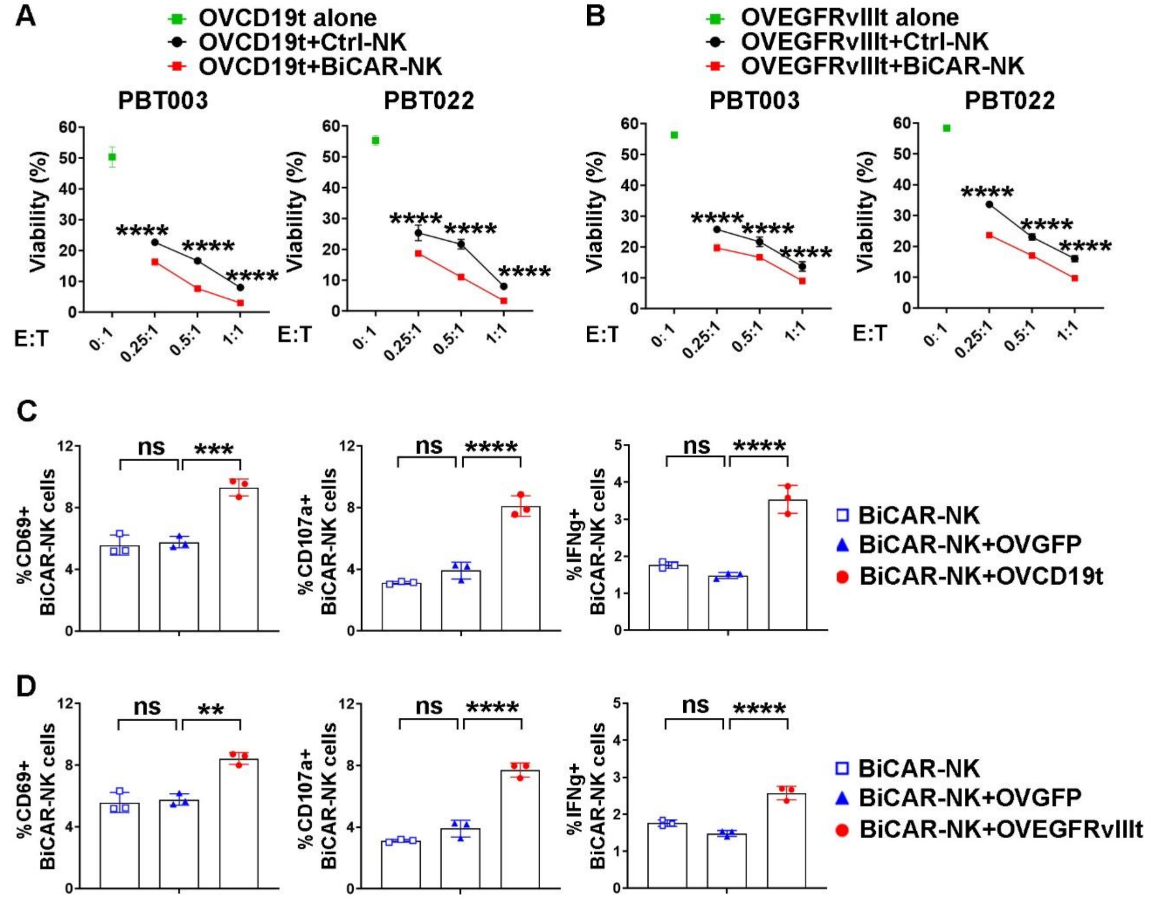

Fig. S12. **BiCAR-NK cells are activated by CD19t- or EGFRvIIIt-encoding oncolytic virus.** (**A** and **B**) Enhanced cytotoxicity of BiCAR-NK cells against OVCD19t-infected GBM cells (A) or OVEGFRvIIIt-infected GBM cells (B). Control (Ctrl)-NK or BiCAR-NK cells were cocultured with GBM cells (PBT003 and PBT022) infected with OVDual for 12 h, and cytotoxicity was assessed using a luciferase reporter assay. n = 3 cell culture replicates. Data are presented as mean ± SD and were analyzed by two-way ANOVA with Tukey's multiple comparisons test. ****$p < 0.0001$. (**C** and **D**) Enhanced activation of BiCAR-NK cells infected with OVCD19t (C) or OVEGFRvIIIt (D) as revealed by an increased percentage of CD69-positive, CD107a positive, or IFNγ-positive cells detected by flow cytometry analysis. Because the experiments for Fig. S12C and S12D were performed together, the BiCAR-NK and BiCAR-NK+OVGFP control groups are shared in these two panels. n = 3 cell culture replicates. Data are presented as mean ± SD and were analyzed by one-way ANOVA with Tukey's multiple comparisons test. **$p = 0.0013$, ***$p = 0.0005$, ****$p < 0.0001$, and ns means no significant difference. Source data are provided as a Source Data file.

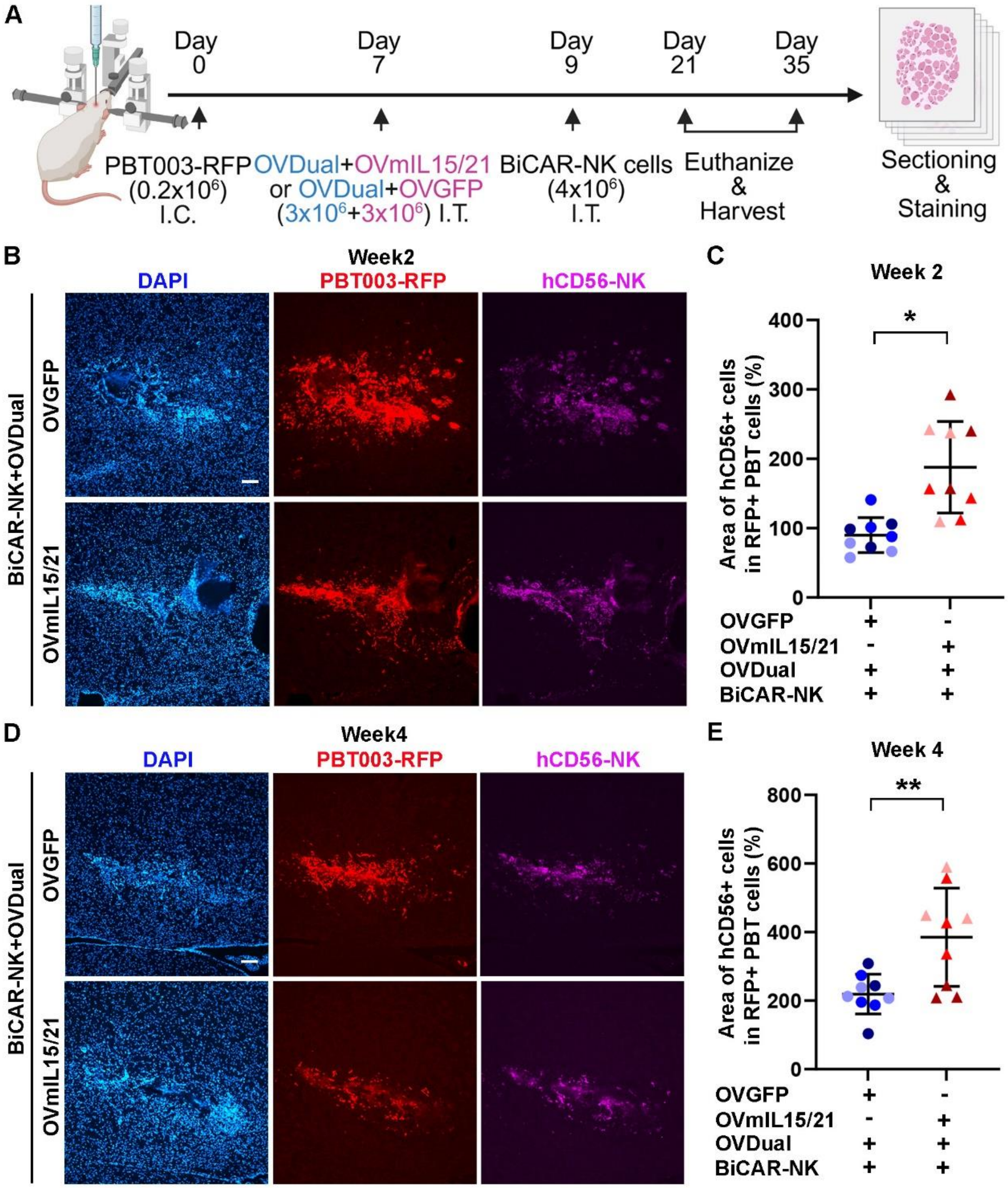

Fig. S13. **OVmIL15/21 promotes BiCAR-NK engraftment and infiltration into tumor tissues in tumor-bearing mouse brains.** (**A**) A schematic showing the experimental procedure (generated using BioRender). (**B** and **D**) Representative images showing BiCAR-NK cells in brain sections from mice received different treatments, detected by anti-hCD56 antibody staining at 2 weeks (B) or 4 weeks (D) after OV treatment. Scale bar, 100 µm. (**C** and **E**) Quantification of the percentage of hCD56-positive NK cell area within the RFP-positive tumor area 2 weeks (C) or 4 weeks (E) after OV treatment. n = 3 mice per group; each color represents three slides from an individual mouse (C and E), three brain slices were averaged to generate a single biological replicate. Data are presented as mean ± SD and were analyzed by two-tailed unpaired t tests. *p = 0.0482, **p = 0.0028. Source data are provided as a Source Data file.

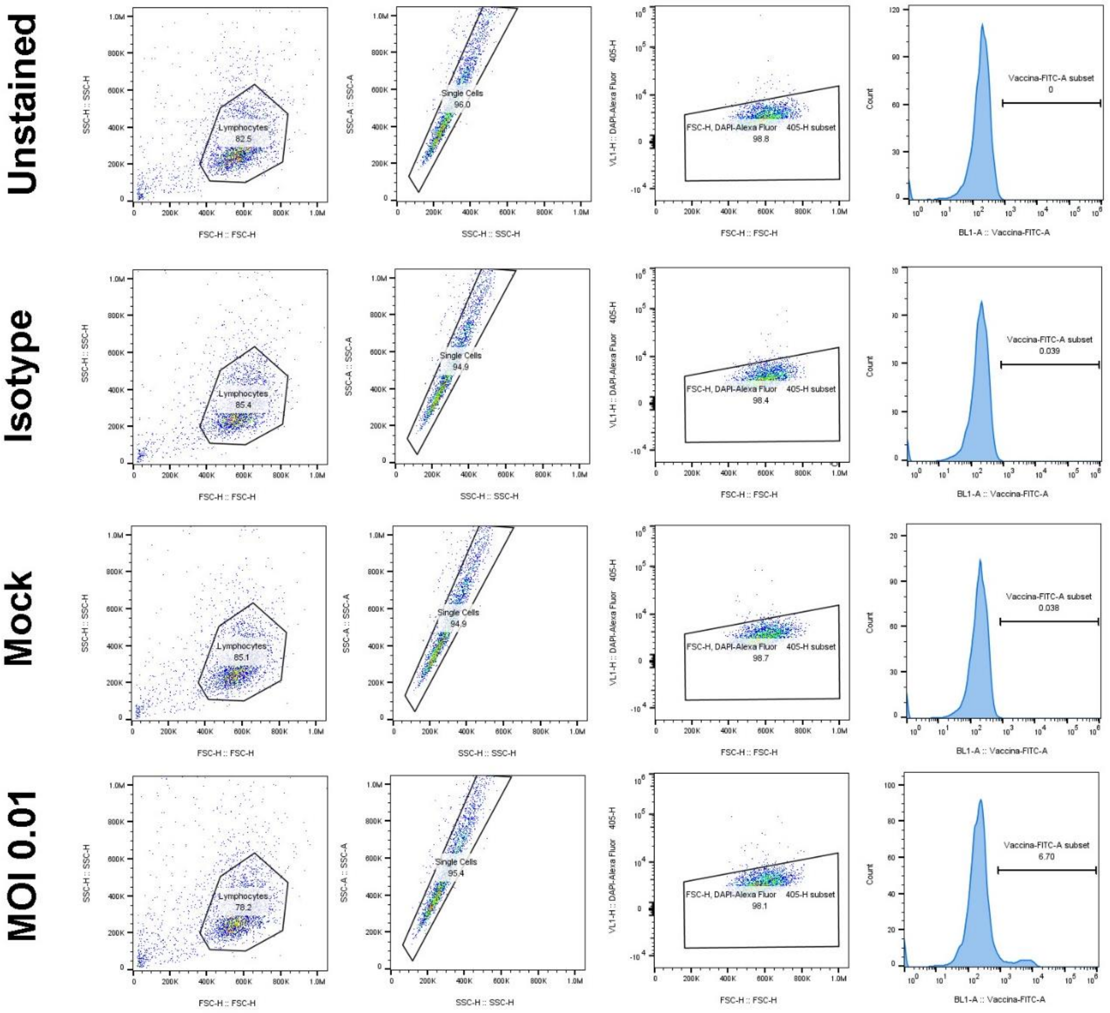

Fig. S14. **Flow cytometry gating strategy.** Representative gating strategy applied to unstained, isotype control, mock-infected, and OV-infected (MOI 0.01) samples. First, lymphocytes were identified based on forward scatter area (FSC-H) versus side scatter height (SSC-H) characteristics. Doublets were excluded by gating on single cells using SSC-A versus SSC-H. Dead cells were excluded by gating to include only DAPI-negative cells (FSC-H vs. DAPI-Alexa Fluor 405-H), and viable singlet cells were retained for downstream analysis. Vaccinia-FITC positivity was then assessed within the live single-cell population using BL1-A (Vaccinia-FITC-A) fluorescence intensity. The unstained and isotype control samples were used to define background fluorescence and establish the positive gate. The percentage of Vaccinia-FITC–positive cells is indicated in each histogram.

Table S1. **Antibodies used in this study**

Antibodies	Clone/ Clonality	Suppliers and Catalog #	Lot #	Dilutions
Anti-hCD19 (APC-conjugated)	SJ25C1	Invitrogen, MHCD1905	2297979	1:50
Anti-hCD19	CAT-13.1E10	Novus, NBP3-12065	T2006B22	1:100
Anti-hEGFRvIII	DH8.3	Novus, NBP2-50599	A-6	1:100
Anti-Vaccinia virus	Polyclonal	Abcam, ab35219	1022881-7	1:200
Anti-hCD34 (PE-conjugated)	581	BD, 550761	8270900	1:50
Anti-hCD3 (FITC-conjugated)	UCHT-1	BD, 555916	3054006	1:50
Anti-hCD45	HI30	Proteintech, 65109-1	51002208	1:800
Anti-hCD45 (PE-conjugated)	HI30	BD, 555483	1075488	1:50
Anti-hCD56	B159	BD, 555518	3304385	1:50
Anti-hIL21	148002	R&D, MAB1500-100	CLBA0122071	1:200
Anti-hIL21 (APC-conjugated)	3A3-N2.1	BD, 560493	1292898	1:50
Anti-hIL15	Polyclonal	R&D, AF315-SP	DJM0222122	1:200
Anti-β-Actin	C4	Santa Cruz, sc-47778	E1721	1:6000
Anti-CD68	FA-11	Fisher Scientific, 501129259	3009288	1:200
Anti-IBA1	Polyclonal	Abcam, ab5076	1029588-4	1:200
Anti-GS Linker (PE-conjugated)	20H7	GenScript, A02314-100	2412K025	1:50
Anti-CD25 (APC-conjugated)	M-A251	BD, 560987	4274623	1:50
Anti-CD69 (PE-conjugated)	FN50	BD, 560968	3327939	1:50
Anti-CD107a (FITC-conjugated)	H4A3	BD, 560949	555800	1:50
Anti-IFN-γ	B27	BD, 5554702	1047222	1:50
Alex 647-conjugated anti-mouse IgG (H+L)	Polyclonal	Jackson ImmunoResearch, 715-605-150	143150	1:100
Alexa 488-conjugated anti-rabbit IgG (H+L)	Polyclonal	Jackson ImmunoResearch, 711-545-152	177621	1:100
Alex 647-conjugated anti-goat IgG (H+L)	Polyclonal	Jackson ImmunoResearch, 705-605-147	177350	1:100
Alexa 488-conjugated anti-rat IgG (H+L)	Polyclonal	Jackson ImmunoResearch, 711-545-152	169269	1:100
Alexa 488-conjugated anti-mouse IgG (H+L)	Polyclonal	Jackson ImmunoResearch, 715-545-150	1631355	1:100
Cy3-conjugated anti-mouse IgG (H+L)	Polyclonal	Jackson ImmunoResearch, 715-165-150	127319	1:100
