# Supplementary material for: Developing a multimodal therapy for glioblastoma using oncolytic virus delivering CD19 and EGFRvIII antigens and bi-specific CARs
Source: Nat Commun. 2026 Apr 9;17:4839. doi: 10.1038/s41467-026-71021-x (PMC13222885; doi:10.1038/s41467-026-71021-x)
Supplement: Supplementary file 2 — Reporting Summary [file 41467_2026_71021_MOESM2_ESM.pdf]

nature portfolio

Corresponding author(s): Yanhong Shi
Last updated by author(s): Feb 12, 2026

Reporting Summary

Nature Portfolio wishes to improve the reproducibility of the work that we publish. This form provides structure for consistency and transparency in reporting. For further information on Nature Portfolio policies, see our Editorial Policies and the Editorial Policy Checklist.

Statistics

For all statistical analyses, confirm that the following items are present in the figure legend, table legend, main text, or Methods section.

n/a	Confirmed	
☐	☒	The exact sample size (*n*) for each experimental group/condition, given as a discrete number and unit of measurement
☐	☒	A statement on whether measurements were taken from distinct samples or whether the same sample was measured repeatedly
☐	☒	The statistical test(s) used AND whether they are one- or two-sided<br>*Only common tests should be described solely by name; describe more complex techniques in the Methods section.*
☒	☐	A description of all covariates tested
☒	☐	A description of any assumptions or corrections, such as tests of normality and adjustment for multiple comparisons
☐	☒	A full description of the statistical parameters including central tendency (e.g. means) or other basic estimates (e.g. regression coefficient) AND variation (e.g. standard deviation) or associated estimates of uncertainty (e.g. confidence intervals)
☐	☒	For null hypothesis testing, the test statistic (e.g. *F*, *t*, *r*) with confidence intervals, effect sizes, degrees of freedom and *P* value noted<br>*Give P values as exact values whenever suitable.*
☒	☐	For Bayesian analysis, information on the choice of priors and Markov chain Monte Carlo settings
☒	☐	For hierarchical and complex designs, identification of the appropriate level for tests and full reporting of outcomes
☒	☐	Estimates of effect sizes (e.g. Cohen's *d*, Pearson's *r*), indicating how they were calculated

Our web collection on statistics for biologists contains articles on many of the points above.

Software and code

Policy information about availability of computer code

Data collection	Bio-Rad's Image Lab 6.0, Spectral Instruments Imaging-AMIView 1.7.061, Zeiss LSM900 microscope, Nikon ECLIPSE Ti2 inverted microscope. Attune NxT flow cytometer (Invitrogen).
Data analysis	Bio-Rad's Image Lab 6.0, Spectral Instruments Imaging-AMIView 1.7.061, Zeiss LSM900 microscope, Nikon ECLIPSE Ti2 inverted microscope, GraphPad Prism 8, Flowjo 10.7.1.

For manuscripts utilizing custom algorithms or software that are central to the research but not yet described in published literature, software must be made available to editors and reviewers. We strongly encourage code deposition in a community repository (e.g. GitHub). See the Nature Portfolio guidelines for submitting code & software for further information.

Data

Policy information about availability of data

All manuscripts must include a data availability statement. This statement should provide the following information, where applicable:

- Accession codes, unique identifiers, or web links for publicly available datasets
- A description of any restrictions on data availability
- For clinical datasets or third party data, please ensure that the statement adheres to our policy

Data availability

The RNA-seq dataset has been uploaded to GEO database with GEO# GSE310003 (https://www.ncbi.nlm.nih.gov/geo/query/acc.cgi). Source data for all figures and supplementary figures are provided within this paper.

Research involving human participants, their data, or biological material

Policy information about studies with human participants or human data. See also policy information about sex, gender (identity/presentation), and sexual orientation and race, ethnicity and racism.

Reporting on sex and gender	Human GBM cells were generated using de-identified GBM patient leftover surgical samples. Specifically, PBT003 cells were derived from a female patient, PBT017 cells were derived from a male patient, PBT022 cells were derived from a male patient, PBT707 cells were derived from a male patient, PBT726 cells were derived from a female patient, and PBT419 cells were derived from a male patient. Human H9 ESCs (WiCell, Cat# WA09) were derived from female human blastocysts. Human PBMCs were isolated from a de-identified male donor.
Reporting on race, ethnicity, or other socially relevant groupings	Information on race, ethnicity, or other socially relevant groups for human cell lines used in this study is not available.
Population characteristics	Healthy blood donors, GBM patients.
Recruitment	N/A. Patients were not recruited for this study. The research used specimens without identifiers obtained from leftover surgical samples or healthy blood donors.
Ethics oversight	Specimens without identifiers from leftover surgical samples and healthy blood donors were used in this study. The information was evaluated and determined to not involve human subjects research by the City of Hope Institute Review Board (IRB).

Note that full information on the approval of the study protocol must also be provided in the manuscript.

Field-specific reporting

Please select the one below that is the best fit for your research. If you are not sure, read the appropriate sections before making your selection.

☒ Life sciences ☐ Behavioural & social sciences ☐ Ecological, evolutionary & environmental sciences

For a reference copy of the document with all sections, see nature.com/documents/nr-reporting-summary-flat.pdf

Life sciences study design

All studies must disclose on these points even when the disclosure is negative.

Sample size	Sample size was determined empirically based on our preliminary data or previous studies. Experiments were performed with sample size n greater than or equal to 3 for statistic analysis.
Data exclusions	For animal study, we excluded mice died prematurely (not due to tumor burden) from analysis.
Replication	Experiments were performed with sample size n greater than or equal to 3 biological replicates, and results from representative experiments were confirmed in at least two independent experiment repeats and in multiple lines of cells.
Randomization	Tumor-bearing mice were randomly grouped into control and treatment groups before treatment in an age-, gender-, and tumor xenogen signal-matched manner between groups. For in vitro experiments, cells were allocated to experimental conditions based on experimental design and treatment conditions. All samples were processed in parallel using identical protocols, reagents, and culture conditions, and comparisons were made only between samples handled concurrently, minimizing technical and biological variability.
Blinding	When monitoring tumor growth, investigators were blinded to the group allocation during the bioluminescence xenogen imaging but were aware of group allocation when assessing the outcome. The investigators were not blinded to group allocation during data analysis to avoid potential mistakes in grouping when analyzing those images. For other experiments, the investigators were not blinded to allocation during experiments and outcome assessment to allow accurate performance and interpretation for the grouping done in the experiments.

Reporting for specific materials, systems and methods

We require information from authors about some types of materials, experimental systems and methods used in many studies. Here, indicate whether each material, system or method listed is relevant to your study. If you are not sure if a list item applies to your research, read the appropriate section before selecting a response.

Materials & experimental systems

n/a	Involved in the study
☐	☒ Antibodies
☐	☒ Eukaryotic cell lines
☐	☐ Palaeontology and archaeology
☐	☒ Animals and other organisms
☐	☐ Clinical data
☐	☐ Dual use research of concern
☐	☐ Plants

Methods

n/a	Involved in the study
☐	☐ ChIP-seq
☐	☒ Flow cytometry
☐	☐ MRI-based neuroimaging

Antibodies

Antibodies used

Anti-human CD19 (APC-conjugated, clone SJ25C1; Invitrogen, MHCD1905; lot 2297979; 1:50); anti-human CD19 (clone CAT-13.1E10; Novus Biologicals, NBP3-12065; lot T2006B22; 1:100); anti-human EGFRvIII (clone DH8.3; Novus Biologicals, NBP2-50599; lot A-6; 1:100); anti-Vaccinia virus (polyclonal; Abcam, ab35219; lot 1022881-7; 1:200); anti-human CD34 (PE-conjugated, clone 581; BD Biosciences, 550761; lot 8270900; 1:50); anti-human CD3 (FITC-conjugated, clone UCHT-1; BD Biosciences, 555916; lot 3054006; 1:50); anti-human CD45 (clone HI30; Proteintech, 65109-1; lot 51002208; 1:800); anti-human CD45 (PE-conjugated, clone HI30; BD Biosciences, 555483; lot 1075488; 1:50); anti-human CD56 (clone B159; BD Biosciences, 555518; lot 3304385; 1:50); anti-human IL-21 (clone 148002; R&D Systems, MAB1500-100; lot CLBA0122071; 1:200); anti-human IL-21 (APC-conjugated, clone 3A3-N2.1; BD Biosciences, 560493; lot 1292898; 1:50); anti-human IL-15 (polyclonal; R&D Systems, AF315-SP; lot DJM0222122; 1:200); anti-β-actin (clone C4; Santa Cruz Biotechnology, sc-47778; lot E1721; 1:6000); anti-CD68 (clone FA-11; Fisher Scientific, 501129259; lot 3009288; 1:200); anti-IBA1 (polyclonal; Abcam, ab5076; lot 1029588-4; 1:200); anti-GS linker (PE-conjugated, clone 20H7; GenScript, A02314-100; lot 2412K025; 1:50); anti-CD25 (APC-conjugated, clone M-A251; BD Biosciences, 560987; lot 4274623; 1:50), anti-CD69 (PE-conjugated, clone FN50; BD Biosciences, 560968; lot 3327939; 1:50), anti-CD107a (FITC-conjugated, clone H4A3; BD Biosciences, 560949; lot 555800; 1:50), and anti-IFN-γ (clone B27; BD Biosciences, 555470; lot 1047222; 1:50).

Validation

All antibodies were commercially available. All primary antibodies used in this study were validated either by the manufacturer for the indicated applications, by prior peer-reviewed literature, and/or by experimental controls included in this manuscript.
Validation details for applications are listed below.
Antibodies used for flow cytometry—including anti-human CD19, CD34, CD3, CD45, CD56, CD25, CD69, CD107a, and IFN-γ—were validated for flow cytometry by the manufacturers as indicated in the corresponding website datasheets. Antibodies used for immunoblotting—including anti-β-actin, anti-human IL-15, and anti-human IL-21 and anti-vaccinia protein—were validated for Western blot applications by the manufacturers and have been widely used in previous studies. In this study, antibody specificity was confirmed by detection of bands at the expected molecular weights and by consistent expression patterns across biological replicates. Antibodies used for immunofluorescence and immunohistochemistry—including anti-CD45, anti-Vaccinia, anti-CD19, anti-EGFRvIII, anti-CD68 and anti-IBA1—were validated for these applications by the manufacturers. In this study, staining patterns were consistent with expected cellular localization of relevant proteins. The anti-Vaccinia virus antibody was validated by the manufacturer and previous studies for detection of vaccinia viral proteins. In this study, specificity was confirmed by positive staining in virus-infected samples and absence of signal in uninfected controls.The anti-CD19 and anti-EGFRvIII antibody was validated by the manufacturer and prior reports for detection of the CD19 and EGFRvIII protein. In this study, specificity was confirmed using CD19- and EGFRvIII-positive tumor cells and appropriate negative controls. The anti-GS linker antibody was validated by the manufacturer for detection of GS linker sequences. In this study, specificity was confirmed by selective detection in engineered CAR-expressing cells and absence of signal in non-transduced controls.

Eukaryotic cell lines

Policy information about cell lines and Sex and Gender in Research

Cell line source(s)

Human GBM cells were derived from specimens without identifiers from leftover surgical samples of GBM patients. Human H9 ESCs were purchased from WiCell. Human PBMCs were isolated from peripheral blood of a de-identified adult male donor obtained from City of Hope blood bank.

Authentication

Human GBM cell lines were directly derived from patient tissues, and no further authentication was performed. Human PBMCs were isolated from an healthy adult male donor, and no further authentication was performed. Human H9 ESCs were purchased from WiCell, and no further authentication was performed.

Mycoplasma contamination

All cultures were confirmed to be free of mycoplasma contamination using the MycoAlert PLUS Mycoplasma Detection Kit (Lonza).

Commonly misidentified lines
(See ICLAC register)

No commonly misidentified cell lines were used.

Palaeontology and Archaeology

Specimen provenance

N/A

Specimen deposition | N/A

Dating methods | N/A

☐ Tick this box to confirm that the raw and calibrated dates are available in the paper or in Supplementary Information.

Ethics oversight | N/A

Note that full information on the approval of the study protocol must also be provided in the manuscript.

Animals and other research organisms

Policy information about studies involving animals; ARRIVE guidelines recommended for reporting animal research, and Sex and Gender in Research

Laboratory animals	Male and female NOD/SCID/IL-2rg (NSG) mice, 8-20 weeks of age, were produced in the Animal Resources Center at City of Hope and used in all experiments described in this study. All animal procedures were approved by the City of Hope Institutional Animal Care and Use Committee under protocol number 24025. Mice were housed under controlled conditions (temperature 20–24 °C, humidity 30–70%) with a 12 h light/dark cycle. Animals were euthanized upon reaching predefined humane endpoints, including moribund condition, neurological deficits, or significant weight loss (≥20%).
Wild animals	No wild animals were used.
Reporting on sex	Sex was not considered as a biological variable in this study. The experiments were designed to evaluate tumor growth and therapeutic response in an immunodeficient xenograft model, and no sex-specific effects have been reported for these endpoints in NSG mice. Accordingly, animals were not stratified by sex for allocation or analysis.
Field-collected samples	No field-collected samples were used.
Ethics oversight	All animal-related work was performed under the IACUC protocol 24025, approved by the City of Hope Institutional Animal Care and Use Committee.

Note that full information on the approval of the study protocol must also be provided in the manuscript.

Clinical data

Policy information about clinical studies

All manuscripts should comply with the ICMJE guidelines for publication of clinical research and a completed CONSORT checklist must be included with all submissions.

Clinical trial registration	N/A
Study protocol	N/A
Data collection	N/A
Outcomes	N/A

Dual use research of concern

Policy information about dual use research of concern

Hazards

Could the accidental, deliberate or reckless misuse of agents or technologies generated in the work, or the application of information presented in the manuscript, pose a threat to:

No	Yes	
☒	☐	Public health
☒	☐	National security
☒	☐	Crops and/or livestock
☒	☐	Ecosystems
☒	☐	Any other significant area

Experiments of concern

Does the work involve any of these experiments of concern:

No	Yes	
☒	☐	Demonstrate how to render a vaccine ineffective
☒	☐	Confer resistance to therapeutically useful antibiotics or antiviral agents
☒	☐	Enhance the virulence of a pathogen or render a nonpathogen virulent
☒	☐	Increase transmissibility of a pathogen
☒	☐	Alter the host range of a pathogen
☒	☐	Enable evasion of diagnostic/detection modalities
☒	☐	Enable the weaponization of a biological agent or toxin
☒	☐	Any other potentially harmful combination of experiments and agents

Plants

Seed stocks	N/A
Novel plant genotypes	N/A
Authentication	N/A

ChIP-seq

Data deposition

☐ Confirm that both raw and final processed data have been deposited in a public database such as GEO.

☐ Confirm that you have deposited or provided access to graph files (e.g. BED files) for the called peaks.

Data access links<br>*May remain private before publication.*	N/A
Files in database submission	N/A
Genome browser session<br>(e.g. UCSC)	N/A

Methodology

Replicates	N/A
Sequencing depth	N/A
Antibodies	N/A
Peak calling parameters	N/A
Data quality	N/A
Software	N/A

Flow Cytometry

Plots

Confirm that:

☒ The axis labels state the marker and fluorochrome used (e.g. CD4-FITC).

☒ The axis scales are clearly visible. Include numbers along axes only for bottom left plot of group (a 'group' is an analysis of identical markers).

☒ All plots are contour plots with outliers or pseudocolor plots.

☒ A numerical value for number of cells or percentage (with statistics) is provided.

Methodology

Sample preparation	ESCs and GBM cells were dissociated with Accutase (Gibco, Cat# A1110501) to obtain single-cell suspension for staining. BiCAR-T cells derived from PBMCs were directly used for staining. DPBS containing 2% FBS (Sigma Aldrich, Cat# A7906) was used as the Flow Cytometry Staining buffer. For staining, cells were suspended and incubated with 50 µL of diluted antibodies for 30 min on ice. The detailed information of all the antibodies used was listed in Extended Table S1. For vaccinia viral protein staining, cells were fixed and permeabilized using the BD Cytofix/Cytoperm Fixation/Permeabilization Solution Kit according to the manufacturer's protocol (BD Biosciences, Cat# 554714). Cells were then incubated with anti-vaccinia virus primary antibody (Abcam, Cat# ab35219) for 30 min on ice. Cells were washed twice and then stained with Alexa 488-conjugated anti-rabbit IgG (H+L) secondary antibody (Jackson ImmunoResearch, Cat# 711-545-152) for 30 min on ice. Cells were then washed twice, resuspended in FACS buffer, and analyzed on Attune NxT flow cytometer (Invitrogen). Data were analyzed using the FlowJo software. Gates were created based on unstained control or isotype control for each sample.
Instrument	Attune NxT Flow Cytometer, which is made by Thermo Fisher Scientific; BD FACSAria™ III Cell Sorter, which is made by BD Biosciences.
Software	Data were analyzed using FlowJo 10.7.1.
Cell population abundance	The abundance of all BiCAR-T cell populations related to fluorescence-activated cell sorting (FACS) has been presented in Figure 1 of the manuscript. PBMCs transduced with BiCAR-carrying lentivirus were stimulated with CD3/CD28 beads, then co-incubated with an anti-hCD34 and anti-G4S linker antibody, followed by washing and loading for flow sorting. More than 90% cells were BiCAR-positive. FACS of ESCs was done similar to FACS of PBMC. H9 ESCs were transduced with lentivirus encoding BiCAR with truncated CD34 as a tag. The transduced cells were cultured for at least two passages and then subjected to FACS. Clonal CAR-positive cells were selected and expanded in mTeSR plus medium on Matrigel-coated plates and banked for subsequent differentiation. More than 90% cells were BiCAR-positive. These cells were used for NK differentiation and further experiments.
Gating strategy	Cells were gated to exclude cell debris and doublets, and then followed by exclusion of dead cells by gating on DAPI negative cells.

☒ Tick this box to confirm that a figure exemplifying the gating strategy is provided in the Supplementary Information.

Magnetic resonance imaging

Experimental design

Design type	N/A
Design specifications	N/A
Behavioral performance measures	N/A

Acquisition

Imaging type(s)	N/A
Field strength	N/A
Sequence & imaging parameters	N/A
Area of acquisition	N/A

Diffusion MRI ☐ Used ☒ Not used

Preprocessing

Preprocessing software	N/A

Normalization	N/A
Normalization template	N/A
Noise and artifact removal	N/A
Volume censoring	N/A

Statistical modeling & inference

Model type and settings	N/A
Effect(s) tested	N/A

Specify type of analysis: ☐ Whole brain ☐ ROI-based ☐ Both

Statistic type for inference (See Eklund et al. 2016)	N/A
Correction	N/A

Models & analysis

n/a	Involved in the study
☒	☐ Functional and/or effective connectivity
☒	☐ Graph analysis
☒	☐ Multivariate modeling or predictive analysis
